# Characterization and Computation of Yb/TiO_2_ and Its Photocatalytic Degradation with Benzohydroxamic Acid

**DOI:** 10.3390/ijerph14121471

**Published:** 2017-11-28

**Authors:** Xianping Luo, Sipin Zhu, Junyu Wang, Chunying Wang, Min Wu

**Affiliations:** 1 Faculty of Resource and Environmental Engineering, Jiangxi University of Science and Technology, Ganzhou 341000, China; lxp9491@163.com (X.L.); Zhusipin926@163.com (S.Z.); Wangjunyu1025@163.com (J.W.); 2 Post-Doctoral Scientific Research Workstation of Western Mining Co. Ltd., Xining 810001, China; wumin5836@163.com; 3 Jiangxi Key Laboratory of Mining and Metallurgy Environmental Pollution Control, Ganzhou 341000, China

**Keywords:** benzohydroxamic acid, characterization, computation, rare earth, Yb, active species

## Abstract

Yb-doped TiO_2_ (Yb/TiO_2_) compositions were synthesized by sol-gel method, and the prepared materials were characterized by X-ray Diffraction (XRD), X-ray photoelectron spectroscopy (XPS), UV-visible diffuse-reflectance spectrum (UV-Vis DRS), transmission electron microscope (TEM) and high resolution transmission electron microscope (HRTEM), energy dispersive spectrometer (EDS), and N_2_ adsorption. A beneficiation reagent of benzohydroxamic acid (BHA) was used to test the photocatalytic activity of Yb/TiO_2_. The characterizations indicate that the doping of Yb could inhibit the crystal growth of TiO_2_, enhance the specific surface area, increase the binding energy of Ti2p, and also slightly expand the adsorption ranges to visible light. Furthermore, the computation of band structure also indicates that Yb-doped TiO_2_ could make the forbidden band narrower than pure anatase TiO_2_, which presents a red shift in the absorption spectrum. As a result of the photodegradation experiment on BHA, Yb/TiO_2_ (0.50% in mass) sintered at 450 °C displayed the highest catalytic activity for BHA when compared with pure TiO_2_ or other doped Yb/TiO_2_ compositions, and more than 89.2% of the total organic carbon was removed after 120 min. Almost all anions, including Cl^−^, HCO_3_^−^, NO_3_^−^, and SO_4_^2−^, inhibited the degradation of BHA by Yb/TiO_2_, and their inhibition effects followed the order of HCO_3_^−^ > NO_3_^−^ > SO_4_^2−^ > Cl^−^. Cations of Na^+^, K^+^, Ca^2+^, and Mg^2+^ displayed a slight suppressing effect due to the impact of Cl^−^ coexisting in the solution. In addition, Yb/TiO_2_ maintained a high photocatalytic ability with respect to BHA after four runs. It is hypothesized that ·OH is one of the main species involved in the photodegradation of BHA, and the mutual transformation of Yb^3+^ and Yb^2+^ could promote the separation of electron-hole pairs.

## 1. Introduction

TiO_2_ is considered to be one of the most promising and widely used semiconductor photocatalyst materials, primarily due to its chemical stability, unique optical properties, nontoxicity, strong oxidizing power, and low cost [1]. However, the largest obstacles to its use are the wide band gap (3.2 eV) and the easy recombination of photogenerated electron-hole pairs, which limit the use of solar light and reduce photon efficiency [2]. Therefore, improvement of photocatalytic activity has been a core topic in photocatalysis research field in recent years. Doping with metal ions in the TiO_2_ lattice has been proven to be an efficient way to enhance photocatalytic activity [3,4]. Research has shown that the doping of rare earth ions can effectively improve the photocatalytic activity of TiO_2_ [5,6,7]. Rare earth elements have the electronic structure of 4f, which exists at the ground and excited states. When photo-excited electrons were captured, Re^3+^ changed into Re^2+^. After that, Re^2+^ was able to react with O_2_ adsorbed on the surface of catalysts to produce Re^3+^ and O_2_^−^. Thus the doping of rare earth could reduce the recombination of photogenerated electron-hole pairs [8,9].

Benzohydroxamic acid (BHA) is most widely used as a collector in the flotation industry at present. However, the dosage of BHA is usually more than 1 kg/t ore [10]. The discharge of mineral-processing wastewater containing BHA from concentrator plants can increase COD content and result in eutrophication because of the benzene ring structure and accumulation of N elements [11]. Besides, the degradation process of BHA is complex, and it can remain for several years or even longer in water. Finally, BHA is be harmful to the water environment and needs to be treated. The biodegradation method has been used to treat BHA [12]. However, five or more days were needed to obtain a removal efficiency of 85% under the conditions of additional nutrition. Our previous study indicated that La/TiO_2_ possessed high activity with respect to BHA [13]. However, the products of BHA and the oxidation mechanisms were not discussed in the previous study.

In this study, Yb-doped TiO_2_ (Yb/TiO_2_) composites were synthesized by the sol-gel method, and their physicochemical properties were characterized by XRD, X-ray photoelectron spectroscopy (XPS), UV-visible diffuse-reflectance spectrum (UV-Vis DRS), N_2_ adsorption, and TEM. Besides, the band structure and density of states were calculated by computer. As a target pollutant, BHA was used to test the photocatalytic activity of the Yb/TiO_2_ composition. The effects of Yb-doping amount, calcination temperature, common anions and cations in the photodegradation of BHA, and the degradation mechanism of BHA were investigated, and the reuse of doped catalyst was also investigated. 

## 2. Materials and Methods

### 2.1. Materials

Tetra butyl titanite and Ytterbium nitrate were bought from the National Medicine Group Chemical Reagent Co. Ltd., Shanghai, China. Anhydrous ethanol, KI, ammonium oxalate, acetic acid, and isopropanol were obtained from the Tianjin Damao Chemical Reagent Factory, Tianjin, China. Benzohydroxamic acid was bought from Shanghai EKEAR Bio@Tech Co. Ltd., Shanghai, China. All the reagents were of analytical grade and used as received without any purification.

### 2.2. Synthesis of Yb/TiO_2_

Pure TiO_2_ and Yb/TiO_2_ catalysts were synthesized by sol-gel method [13,14,15]. The detailed prepared processes are as follows. First, 10 mL of tetra butyl titanite were dissolved in 15 mL anhydrous ethanol to get a mixture solution noted as solution A, and solution A was stirred for 30 min; second, solution B was prepared using 2 mL acetic acid, 10 mL ethanol, 2 mL deionized water, and Ytterbium nitrate in the required stoichiometry (the doping ratios of Yb were set to 0%, 0.10%, 0.30%, 0.50%, 0.70%, and 1% according to the mass fraction of Yb to TiO_2_.); third, the transparent homogeneous sol was gained by adding dropwise solution B to the solution A under acute and continuous agitation; fourth, the aerogel was formed after ageing at room temperature for 2 days and then dried at 90 °C; and finally, pure TiO_2_ and Yb/TiO_2_ were achieved after calcining (400 °C, 450 °C and 500 °C) of the aerogel in muffle furnace under air atmospheres.

### 2.3. Characterization

The XRD patterns were carried out with an X-ray diffractometer (RigakU Miniflex, Rigaku Corporation, Tokyo, Japan) using monochromatized Cu Kα radiation (*λ* = 0.15418 nm). The element constitutions were analyzed by X-ray photoelectron spectroscopy (XPS) on a Thermo-type multi-function imaging electronic spectrometer (ESCALAB 250XI, Thermo Fisher Scientific, Waltham, MA, USA). The property of light absorption of materials was studied using a UV-Vis spectrophotometer (UV-2550, Shimadzu Scientific Instruments, Tokyo, Japan). Nitrogen adsorption–desorption isotherms were recorded by adsorption apparatus (ASAP 2460, Micromeritics Instrument Corporation, Norcross, GA, USA) at liquid nitrogen temperature (−196 °C) and the specific surface area and average pore size of TiO_2_ or La/TiO_2_ were measured by the Brunauer-Emmett-Teller (BET) method. Morphology of catalysts was observed by a Tecnai transmission electron microscope (FEI G2-20, Hillsboro, AL, USA). 

### 2.4. Photocatalytic Degradation Tests

Band structure and partial density of states (PDOS) were calculated based on the density functional theory (DFT) implemented in the Cambridge Serial Total Energy Package (CASTEP, MS2016) [16].

The general gradient approximation (GGA) exchange–correlational functional was used and treated with Perdew-Burke-Ernzerhof (PBE) scheme. Geometry optimization was adopted. The detailed calculation conditions are as follows: convergence of energy change per atom at 5 × 10^−6^ eV, residual force at 0.01 eV/Å, stress at 0.02 GPa, and displacement of atoms at 0.0005 Å. The Broyden-Fletcher-Goldfarb-Shanno (BFGS) algorithm was used in the optimization. The self-consistent field tolerance was 1.0 × 10^−7^ eV/atom. The FFT (Fast Fourier Transformation) grid of the basis was 50 × 50 × 64 and the kinetic energy cutoff for the plane-waves basis was 400 eV. A 3 × 3 × 3 k-point and the ultrasoft pseudopotential were applied in above calculation. The electronic configurations considered are 3s^2^3p^6^3d^2^4s^2^ (Ti), 2s^2^2p^4^ (O), and 4f^14^ 5s^2^5p^6^6s^2^ (Yb) for TiO_2_ (unit cell) and TiO_2_ (Yb, 2 × 2 × 1 supercell) (Figure S1).

### 2.5. Photocatalytic Degradation Tests

The photocatalytic tests were carried out in a light chemical reaction apparatus from XPA-7 (Xujiang Electromechanical Factory, Nanjing, China). In order to ensure the adsorption-desorption equilibrium of BHA in photocatalysts, the reaction solution was magnetically stirred for 30 min without irradiation. Then, a 300 W mercury light was turned on. Approximately 5 mL of suspension solution was taken at given time intervals and the supernatant was tested by UV-Vis spectrophotometry at 229 nm after being centrifuged. The removal efficiency (*R*) was calculated by Equation (1), where *C*_0_ (mg/L) is the initial concentration of BHA and *C_t_* (mg/L) is the concentration at reaction time *t* (min).

*R* = (*C*_0_ − *C_t_*) × 100%/*C*_0_ (1)

The total organic carbon (TOC) was detected by the total organic carbon analyzer (Elementar Analysensysteme GmbH, Langenselbold, Germany). Ionic chromatography (ICS-1100, Thermo Fisher Scientific, Waltham, MA, USA) was used to detect the existence and concentration of NO_3_^−^ and NO_2_^−^ in degradation products. In addition, the effects of common inorganic cations and anions (Na^+^, K^+^, Ca^2+^, Mg^2+^, Cl^−^, HCO_3_^−^, NO_3_^−^, SO_4_^2−^) in waters on the photodegradation of BHA were investigated. 

## 3. Results and Discussion

### 3.1. Characterization

#### 3.1.1. XRD

**Figure 1:**
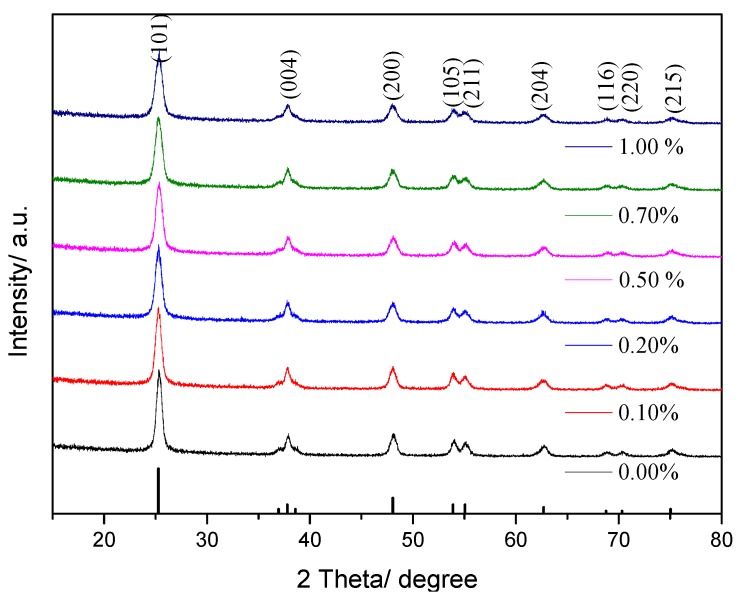
XRD (X-ray Diffraction) patterns of Yb/TiO_2_ with different doping amounts, calcined at 450 °C.

Figure 1 shows the XRD patterns of prepared TiO_2_ doped with different amounts of Yb. It is observed that major peaks at 2*θ* values of 25.21°, 37.53°, 47.87°, 53.53°, 54.86°, 62.37°, 68.30°, 70.03° and 74.63° corresponded to the diffraction planes (101), (004), (200), (105), (211), (204), (116), (220) and (215), respectively, of anatase TiO_2_. Hence, Yb/TiO_2_ retains the crystal phase of TiO_2_. However, the diffraction peak of pure TiO_2_ is relatively narrow compared with Yb/TiO_2_. The higher the Yb doping amount, the broader of the peaks, and the lower of the intensity [17]. In other words, the doping of Yb slowed down the growth of TiO_2_ nanoparticles, changed the structure of TiO_2_ particles, and increased the diffusion barrier of grain. Thus, doping of Yb led to a systematic decrease in grain.

The Scherrer formula [18] is used to calculate the crystallite sizes in the form of Equation (2):

*D* = 0.89*λ*/*βcosθ* (2)

where *D* is crystallite size, *λ* (nm) is wavelength of X-ray, *β* (rad) is full width at half maximum of the peak, and *θ* (rad) is the angle of diffraction. As a result, the crystallite sizes of pure TiO_2_ and 0.50% Yb/TiO_2_ are 19 nm and 13 nm, respectively. It is proposed that the doping of Yb could inhibit the crystal growth of TiO_2_ because of quantum size. Liu reported that the RE-doped TiO_2_ nanocrystals almost had the capacity to enhance photocatalytic activity due to the quantum size effect of prepared RE^3+^/TiO_2_ [19]. 

#### 3.1.2. XPS

**Figure 2:**
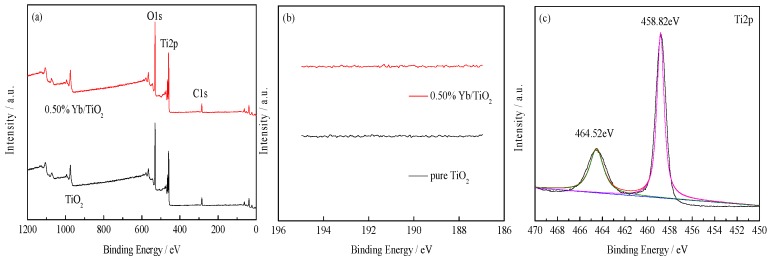

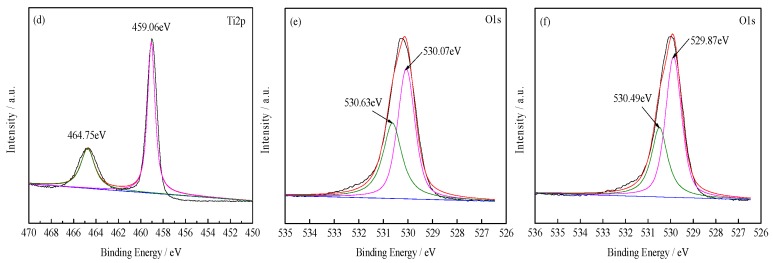
X-ray photoelectron spectroscopy (XPS) spectra. Survey spectra (**a**), Yb3d (**b**), Ti2p and O1s of pure TiO_2_ (**c**,**e**), and Ti2p and O1s of Yb/TiO_2_ (**d**,**f**), respectively. Different color mean different curves in Figure 2c–f. Black, original curve; red, fitted curve; green, hydroxyl oxygen curve; pink, crystal lattice oxygen curve; blue, background.

As seen from Figure 2a of the survey spectrum, there are relative peaks corresponding to the elements Ti and O that come from the prepared TiO_2_. Figure 2b shows the spectra ranges of doped Yb. However, similar to pure TiO_2_, the characteristic peaks of Yb^3+^ and Yb^2+^ are not detected near 182.1 eV and 190.9 eV, which indicates that the content of Yb on the surface of the catalyst was very low.

The two peaks of Ti2p for pure TiO_2_ and Yb/TiO_2_ were composed of Ti2p1/2 and Ti2p3/2, which indicates the existence of a +4 valence for Ti atom. The Ti peaks of pure TiO_2_ are at 464.52 eV and 458.82 eV, while the peaks are at 464.75 eV and 459.06 eV for Yb/TiO_2_. As seen from Figure 2e,f, the characteristic peak of O1s was wide and asymmetric, and was fitted into two kinds of chemical states by curve fitting: crystal lattice oxygen (lower) and hydroxyl oxygen (higher) [1]. The fitted peak of O1s located at 530.07 eV was for lattice oxygen, while the peak at 530.63 eV corresponded to hydroxyl oxygen on the surface of pure TiO_2_. After doping, O1s spectrum peaks slightly decreased to 529.87 eV and 530.49 eV for lattice oxygen and hydroxyl oxygen, respectively. As discussed above, the binding energy of Ti2p increased, while the binding energy of O1s decreased for Yb/TiO_2_ compared pure TiO_2_. This might be due to the fact that Yb and Ti elements combine to form new chemical bonds of Ti-Yb [20], which leads to a decrease of +4 valences for Ti in Yb/TiO_2_.

#### 3.1.3. UV-Vis DRS

**Figure 3:**
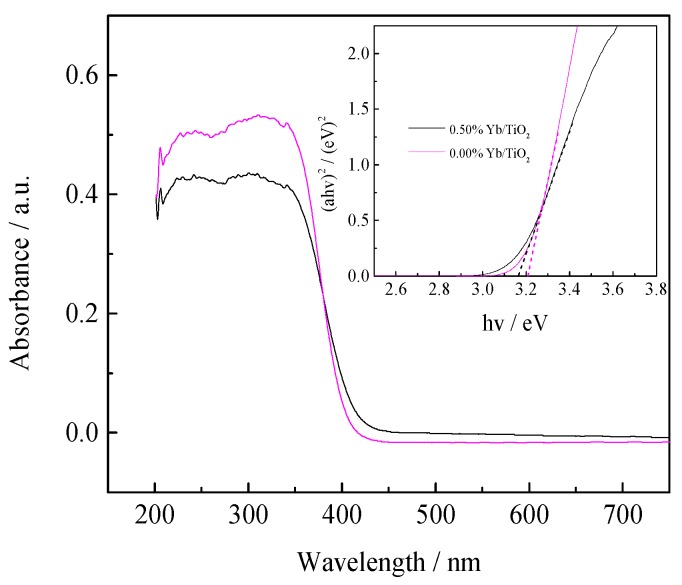
UV-Vis DRS spectra of pure TiO_2_ and 0.50% Yb/TiO_2_. UV-Vis DRS: UV-visible diffuse-reflectance spectrum.

The UV-Vis DRS curves of pure TiO_2_ and 0.50% Yb/TiO_2_ are shown in Figure 3. There is a slight red shift of Yb/TiO_2_ compared with pure TiO_2_, which was supported by the band gap energy (*Eg*) calculated by Formula (3) as follows [21]:

*ahv* = *A* (*hv* − *Eg*)*^n^*^/2^ (3)

where *v*, *h*, *A* and *a* represent the light frequency, Planck’s constant, a constant, and the absorption coefficient, respectively. The value of n is determined by the semiconductor’s optical transition type: for indirect transition, *n* = 4; and for direct transition, *n* = 1. By calculation, the *Eg* values for pure TiO_2_ and 0.50% Yb/TiO_2_ were 3.20 eV and 3.16 eV, respectively. The red shift could be attributed to the charge-transfer transition between the 4f electrons of Yb and the conduction or valence band of TiO_2_ [22]. As discussed above, the doping of Yb can narrow the band gap of TiO_2_ and reduce the band gap energy, which is important to slow down the recombination rate of the electron-hole pairs and ultimately augment the photocatalytic activity [23].

#### 3.1.4. *S*_BET_ and Porosity Analysis

**Figure 4:**
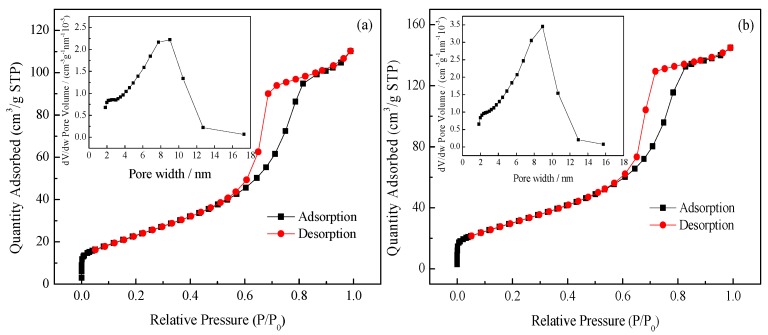
N_2_ adsorption-desorption isotherms and pore size distributions (inset) of pure TiO_2_ (**a**) and 0.50% Yb/TiO_2_ (**b**).

The N_2_ adsorption-desorption isotherms in Figure 4 indicate the typical Langmuir IV isotherm with a hysteresis loop, which indicates the mesoporous structure of synthesized samples [24,25]. The hysteresis loops of pure TiO_2_ and 0.50% Yb/TiO_2_ are consistent with type H_2_ that has uniform particle accumulation in the hole. The shape of the holes is considered to be an ink bottle-shaped channel of the small mouth and large cavity [26]. As shown in Figure 4a,b the distribution of pore size ranges from 2 nm to 10 nm. This is in the mesoporous range and is in accordance with what has been discussed above. Besides, the most probable pore size of pure TiO_2_ was 9.01 nm, while it was 8.94 nm for 0.50% Yb/TiO_2_. The *S*_BET_ values were 87 m^2^/g and 114 m^2^/g for pure TiO_2_ and 0.50% Yb/TiO_2_, respectively. Obviously, the *S*_BET_ of 0.50% Yb/TiO_2_ is much larger than that of pure TiO_2_, which means the doped catalyst could have better adsorption ability than the undoped catalyst. The reason may be due to the linkage between the rare earth ions and titanium by an oxygen bridge, which could effectively enhance the specific surface area of TiO_2_ [27]. Generally speaking, the larger the specific surface area, the more surface reaction sites. This is an advantage for the improvement of photocatalytic activity.

#### 3.1.5. Microstructure Analysis

**Figure 5:**
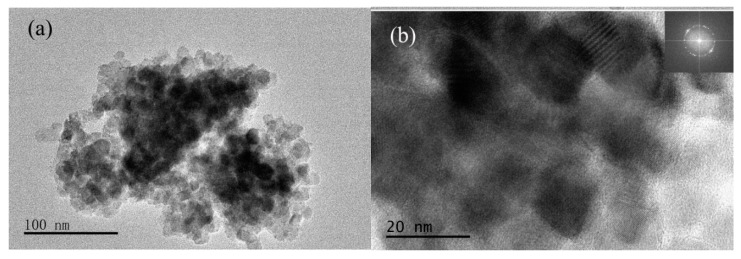
TEM (**a**) and HRTEM (**b**) images of 0.50% Yb/TiO_2_. HRTEM: high resolution transmission electron microscope.

The particle size of 0.50% Yb/TiO_2_ is in the range of 10~15 nm as seen from Figure 5a, which is in accordance with the XRD calculation using the Scherrer formula. However, there is still a significant agglomeration for the doped catalyst due to the smaller particle size. The corresponding FFT pattern formed in Figure 5b shows that the Yb^3+^-doped TiO_2_ is a polycrystalline structure. The compositions and elements of Yb/TiO_2_ are further investigated with EDS (energy dispersive spectrometer): the detected doped amount of Yb was about 0.3%, which was lower than the experimental value of 0.5%, and element mapping indicated successful doping as shown in Figure S2. 

### 3.2. Photodegradation of Benzohydroxamic Acid

#### 3.2.1. Effect of the Doped Amount

The photodegradation of BHA can be described by an apparent first-order equation with a simplified Langmuir-Hinshelwood model: ln(*C*_0_/*C_t_*) = *kt*, where *C*_0_ (mg/L) is the initial concentration of BHA, *C_t_* (mg/L) is the concentration at reaction time *t* (min), and *k* (min^−1^) is the apparent first-order rate constant. Table 1 indicates the linear relationship between ln(*C*_0_/*C_t_*) and *t* (min), confirming that the photodegradation reaction is indeed a first-order reaction. Besides, the rate constant of 0.50% Yb/TiO_2_ was up to 0.0392 min^−1^. Due to the fact that the radius of Ti^4+^ (0.068 nm) is much smaller than that of Yb^3+^ (0.115 nm), Ti^4+^ may enter the lattices of Yb^3+^ during synthesis, which might induce a great deal of lattice distortions and plenty of defects at/under the surface of Yb-doped TiO_2_ nanoparticles [28]. The generated defects would decrease the recombination of electron–hole pairs. However, if the doped amount was continuously increased, this would produce defects, which would become the center of recombination again. Therefore, there exists an optimum doping ratio. It might be 0.5% in this study. As seen from Figure S3, 450 °C was the appropriate prepared temperature, and the following photodegradation experiments all used 0.50% Yb/TiO_2_ calcined at 450 °C.

In addition to photoactivity, reusability is also an important characteristic of photocatalysts. Therefore, four cycling tests were performed to demonstrate the reusability of Yb/TiO_2_ for photodegradation of BHA. As shown in Figure S4, after four cycling tests, the degradation efficiency only reduced about 2% compared to the first run. Yb/TiO_2_ retains photocatalytic activity on photodegradation of BHA, confirming potential reusability in practical applications.

#### 3.2.2. Effect of Inorganic Ions

**Figure 6:**
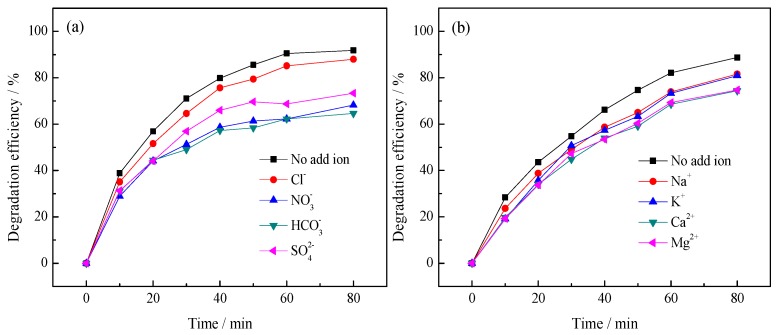
Effect of inorganic anions (**a**) and cations (**b**) on the degradation of benzohydroxamic acid by Yb/TiO_2_. The benzohydroxamic acid (BHA) concentration was 30 mg/L, the catalyst dosage was 0.3 g/L, and the concentrations of cation ions and anion ions were 5 mmol/L.

There are some common inorganic ions (Cl^−^, HCO_3_^−^, NO_3_^−^, SO_4_^2−^, Na^+^, K^+^, Ca^2+^ and Mg^2+^ are taken into account) in natural water. Their existence might affect the removal efficiency of pollutants and Figure 6 illustrates the effects of these eight ions on the photodegradation of BHA by Yb/TiO_2_.

As shown in Figure 6a, Cl^−^ has slight effect on the degradation of BHA. SO_4_^2−^ can react with the valence band hole (h^+^) [29], and compete with BHA for the holes, reducing the degradation rate. However, generated h^+^ is not the main species involved in the photodegradation of BHA, as discussed later. Therefore, SO_4_^2−^ also has little effect on the photodegradation. For NO_3_^−^, Sörensen [30] indicated that NO_3_^−^ could act as an “inner filter” and reduce the UV light intensity to the reaction solution. Therefore, addition of NO_3_^−^ inhibited the removal efficiency of BHA in the reaction process. In addition, nitrate ions were produced as the reaction went on because of the existence of N-containing functional groups in the molecule of BHA. The excessive NO_3_^−^ could be adsorbed on the catalyst and compete for the active sites on the catalyst surface with BHA. As discussed in a published paper [13], addition of HNO_3_ would inhibit the photodegradation significantly, because of the existence of N-containing functional groups in molecular BHA. The detected result by ionic chromatography also manifested in the production of NO_3_^−^ during the photodegradation of BHA. HCO_3_^−^ could react with ·OH to produce carbonate radicals (HCO_3_^−^ + ·OH →·CO_3_^−^ + H_2_O) [31], which possess too weak an oxidizing ability to react with other pollutant molecules. In addition, the later discussion will demonstrate the strong ability of ·OH. Like NO_3_^−^, HCO_3_^−^ displayed significant inhibition effect on BHA photodegradation by Yb/TiO_2_.

As shown in Figure 6b, the four metal ions (Na^+^, K^+^, Ca^2+^, and Mg^2+^) displayed inhibition effects on BHA removal. This is attributed to the effect of Cl^−^ ions co-present in the solution, because the four metal ions were used in their chloride salts. As described above, Cl^−^ ions inhibit photodegradation slightly [32].

### 3.3. Mechanism Analysis

**Figure 7:**
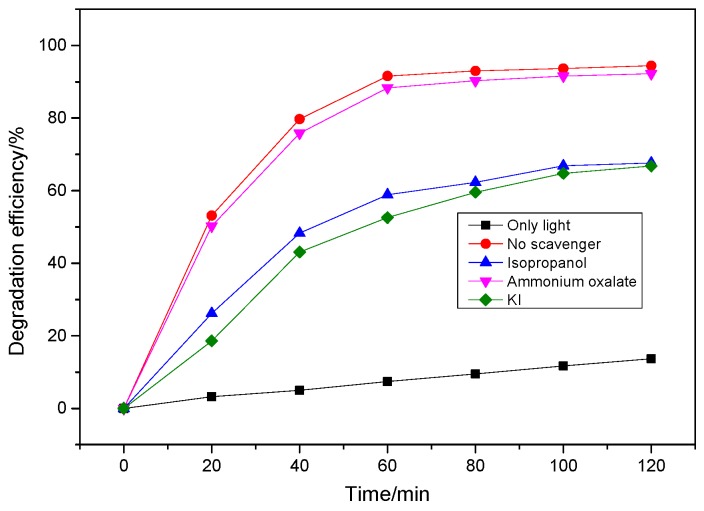
The effect of a series of scavengers on the photocatalytic efficiency of 0.50% Yb/TiO_2_. The BHA concentration was 30 mg/L, the catalyst dosage was 0.5 g/L, and the concentration of all scavengers was 10 mmol/L.

As is well known, active species such as h^+^ and ·OH might play important roles in the photocatalytic oxidation process. In order to reveal the possible photocatalytic mechanism of Yb/TiO_2_ on BHA, the active species trapping experiment were carried out (Figure 7). Isopropanol and ammonium oxalate were used as the scavengers of ·OH and h^+^, respectively [33,34]; KI could not only capture ·OH, but also h^+^ [33]. As a result, the presence of ammonium oxalate (an h^+^ scavenger) almost had no influence on the rate of BHA degradation, while the addition of isopropanol (an ·OH scavenger) and KI (an ·OH and h^+^ scavenger) inhibited the photodegradation of BHA by Yb/TiO_2_. The results suggest that ·OH is one of the main species involved in the photodegradation of BHA.

**Figure 8:**
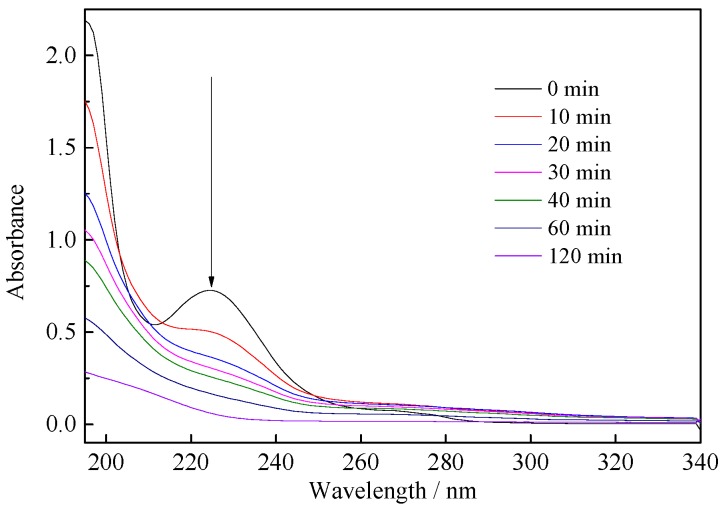
UV-Vis absorbance spectra of benzohydroxamic acid solution at different irradiation times.

UV-Vis absorbance spectra of BHA solutions at different irradiation times were performed to investigate the degradation processes of the beneficiation reagent. As shown in Figure 8, the maximum adsorption intensity of BHA is gradually decreasing with the irradiation time. After 120 min, the light adsorption curve almost becomes a horizontal line, indicating the complete decomposition of BHA by Yb/TiO_2_ during the degradation processes. Besides, the mineralization rate is also an important indicator to measure the degree of degradation of organic pollutants. The removal efficiency of TOC reached 89.2% by 0.50% Yb/TiO_2_ after 120 min of irradiation, while the removal efficiency was 69.5% by pure TiO_2_ (Figure S5). As such, TiO_2_ doped with Yb indeed thoroughly promotes photodegradation.

**Figure 9:**
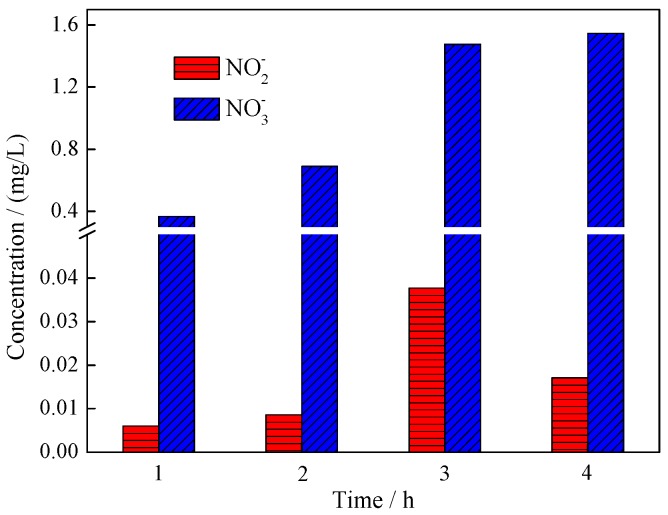
Changes in NO_3_^−^ and NO_2_^−^ concentrations in the photocatalytic degradation of benzohydroxamic acid by 0.50% Yb/TiO_2_.

The TOC data explain the conversion of the C element from organic chemicals to inorganic matter. In addition to the C element, there is also N-containing functional group (amino group) in the molecule of BHA. Both NO_3_^−^ and NO_2_^−^ are detected in the degradation solution at different times (as seen in Figure 9). The concentration of NO_2_^−^ was very low, and the highest concentration of NO_2_^−^ was reached in the reaction at 2 h. However, the concentration of NO_3_^−^ enhances with the reaction. The amino group should be converted to NO_2_^−^, then to NO_3_^−^ (-NH→NO_2_^−^→NO_3_^−^).

### 3.4. Calculations and Interfacial Charge Transfer Processes

**Figure 10:**
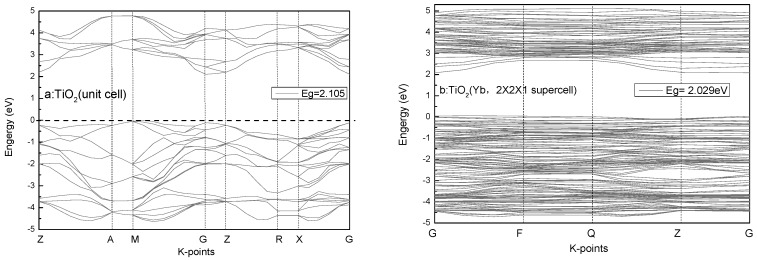
Band-gap structures of pure anatase TiO_2_ and Yb-doped anatase TiO_2_ (2 × 2 × 1 supercell).

The calculated band structure of anatase TiO_2_ (unit cell) exhibits an indirect band gap of 2.105 eV (Figure 10), which is lower than the experimental values, but the electronic structure is otherwise essentially the same [35]. For the doped structure, a supercell of 2 × 2 × 1 48-atom anatase TiO_2_ was used, and one Ti atom near the centre of the supercell was replaced by one Yb atom. The calculated band structure of doped TiO_2_ is 2.029 eV, which is lower than for pure TiO_2_, and is consistent with the experimental results in this study. 

**Figure 11:**
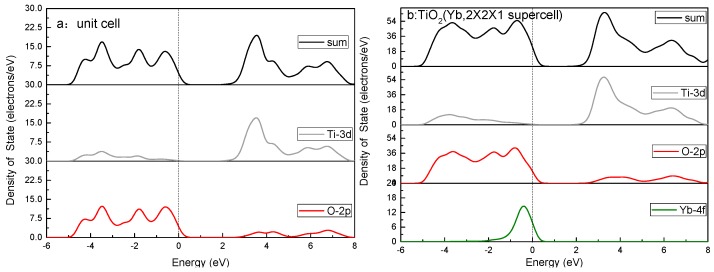
The calculated density of states of pure TiO_2_ and Yb-doped TiO_2_ (2 × 2 × 1 supercell).

It is clear from the screening plots (Figure 11) that the introduced Yb 4f orbital mainly contributes to the top of the valence band, extending the impurities’ energy level into the forbidden band and producing a wider valence band in comparison with the pure cases. Meanwhile, both the top of the valence band and the bottom of the conduction band shift slightly with respect to the Fermi level, which results in the band gap narrowing and the consequent redshift response of the optical absorption edge.

The photocatalytic mechanism of Yb/TiO_2_ may be related to the 4f electronic structure of Yb and its variable valences (+2, +3). Yb^3+^ in Yb/TiO_2_ trapped the photoexcited electrons to translate to Yb^2+^. Yb^2+^ was translated to Yb^3+^ again by release of an electron, and the released electron could be easily captured by O_2_ adsorbed on the surface of TiO_2_ to produce ·O_2_^−^ [9]. On one hand, the process above could inhibit the recombination of photoinduced charge (Equations (4)–(6)); on the other hand, the produced ·O_2_^−^ could enhance the oxidation of BHA (Equation (7)).

Yb^3+^ + e^−^ → Yb^2+^ (4)

Yb^2+^ + O_2_ → Yb^3+^ + ·O_2_^−^ (5)

2·O_2_^−^ + 2H^+^ + e^−^ → ·OH + OH^−^ + O_2_ (6)

·O_2_^−^ + ·OH + Organics (BHA) → Products (7)

## 4. Conclusions

Yb/TiO_2_ composites were prepared, characterized, and calculated by computer. Compared with pure TiO_2_, the XRD diffraction peaks of Yb/TiO_2_ were weaker and broader. The composition doped with Yb could suppress grain growth, enhance the thermal stability of anatase, and increase the *S*_BET_ of TiO_2_. Yb/TiO_2_ also had lower gap energy and more regular shape in morphology than pure TiO_2_. In addition, 0.50% Yb/TiO_2_ (450 °C) indicated higher photocatalytic degradation ability with respect to BHA: the degradation efficiency and mineralization rate of BHA reached 95.5% and 89.2%, respectively, after 120 min with 300 W mercury. The doped Yb could promote the separation of photo-exited electrons and holes, resulting in the increase of the degradation rate of BHA. Moreover, ·OH was the main active species during the photocatalytic process, and NO_3_^−^ was the dominant product of the amino group.

## Figures and Tables

**Table 1:** Parameters of the first-order reaction kinetics.

Doped Ratio	*R*^2^	*k* (min^−1^)
0%	0.9994	0.0339
0.1%	0.9979	0.0375
0.3%	0.9981	0.0372
0.5%	0.9967	0.0392
0.7%	0.9910	0.0335
1%	0.9990	0.0327

## Supplementary Materials

The following are available online at www.mdpi.com/1660-4601/14/12/1471/s1, Figure S1: The unit cell of anatase TiO_2_ (a) and a Yb-doped anatase TiO_2_ supercell (b). The red, gray, and green spheres represent oxygen, titanium, and Ytterbium atoms, respectively; Figure S2: EDS of Yb/TiO_2_ (a). Element mapping images of Ti (b), O (c), and Yb (d) of Yb/TiO_2_; Figure S3: Effect of different calcination temperatures on photocatalytic degradation of benzohydroxamic acid (BHA). The BHA concentration was 30 mg/L, and the catalyst dosage was 0.3 g/L; Figure S4: Recycle tests of Yb/TiO_2_. The BHA concentration was 30 mg/L, and the catalyst dosage was 0.5 g/L; Figure S5: TOC efficiency by 0.50% Yb/TiO_2_, pure TiO_2_, and blank. The BHA concentration was 30 mg/L, and the catalyst dosage was 0.3 g/L.
