# Supplementary material for: Characterization and Computation of Yb/TiO2 and Its Photocatalytic Degradation with Benzohydroxamic Acid
Source: Int J Environ Res Public Health. 2017 Nov 28;14(12):1471. doi: 10.3390/ijerph14121471 (PMC5750890; doi:10.3390/ijerph14121471)
Supplement: Supplementary file 1 [file ijerph-14-01471-s001.pdf]

Supplementary Material

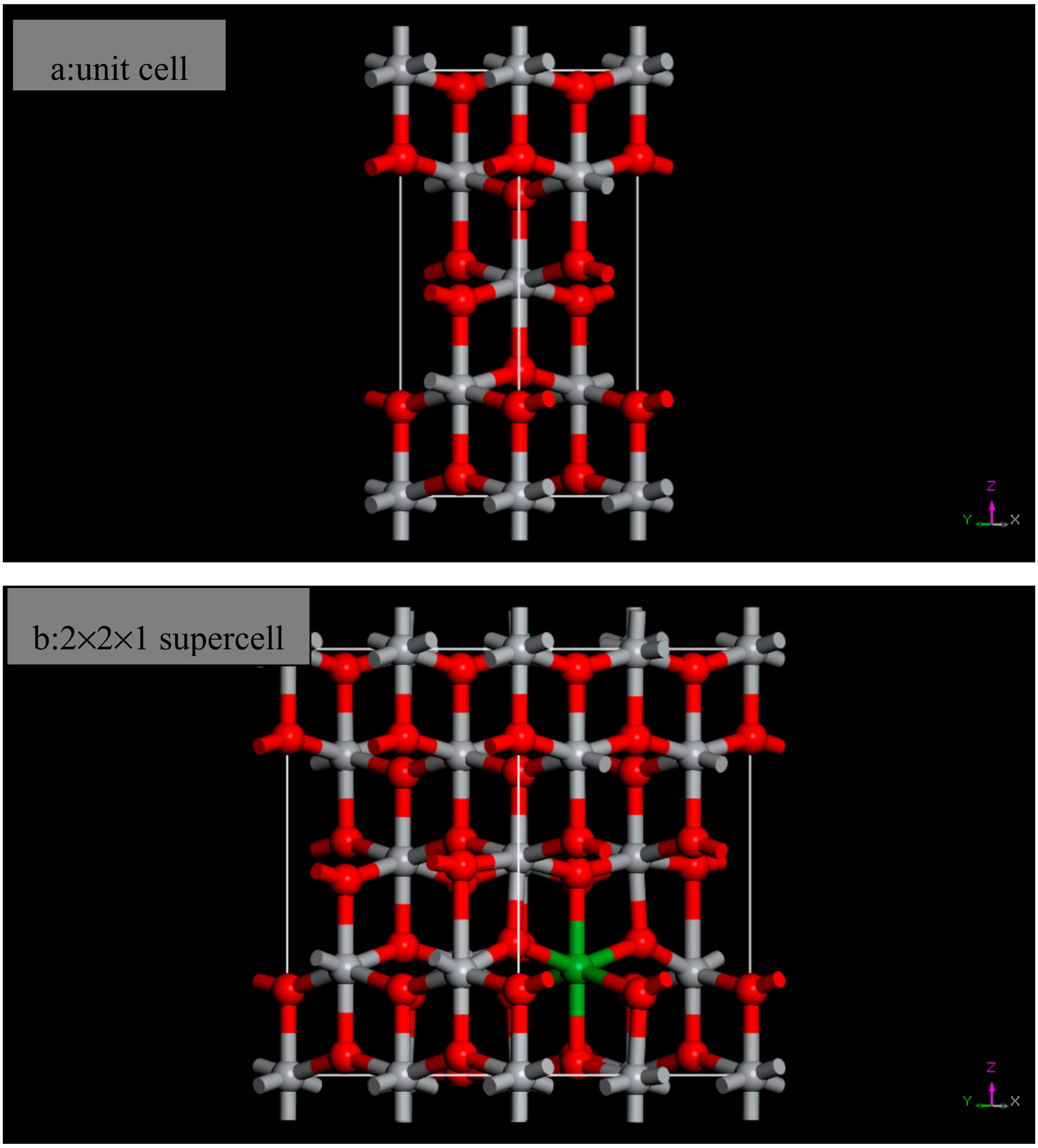

Figure S1. The unit cell of anatase TiO_2 (**a**) and a Yb-doped anatase TiO_2 supercell (**b**). The red, gray, and green spheres represent oxygen, titanium, and Ytterbium atoms, respectively.

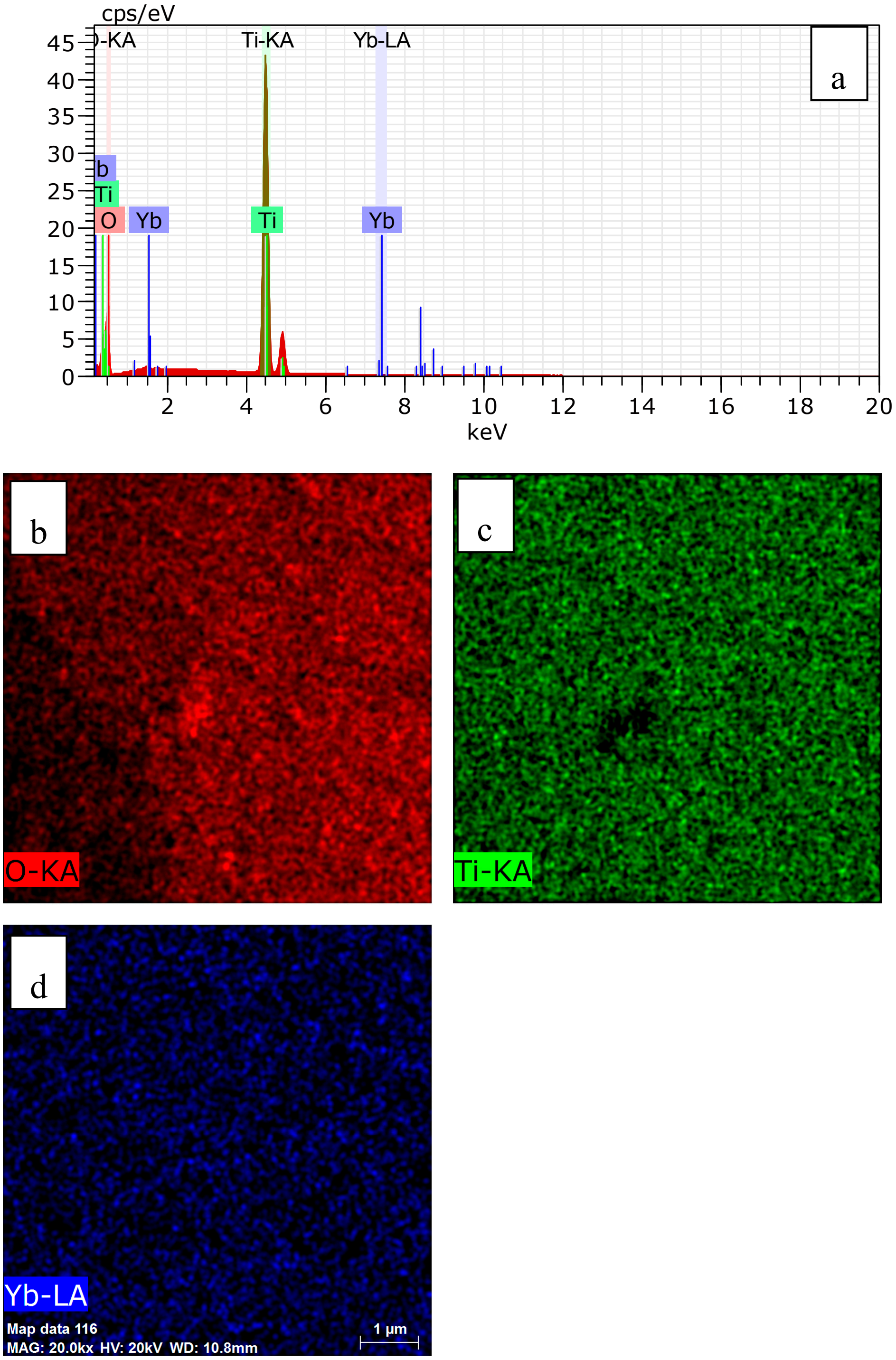

Figure S2. EDS of Yb/TiO_2 (**a**). Element mapping images of Ti (**b**), O (**c**), and Yb (**d**) of Yb/TiO_2. EDS: energy dispersive spectrometer.

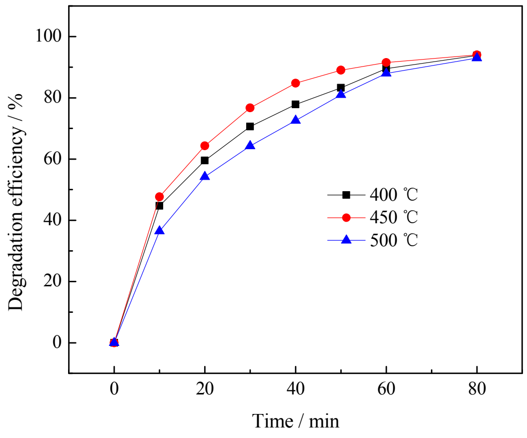

Figure S3. Effect of different calcination temperatures on photocatalytic degradation of benzohydroxamic acid (BHA). The BHA concentration was 30 mg/L, and the catalyst dosage was 0.3 g/L.

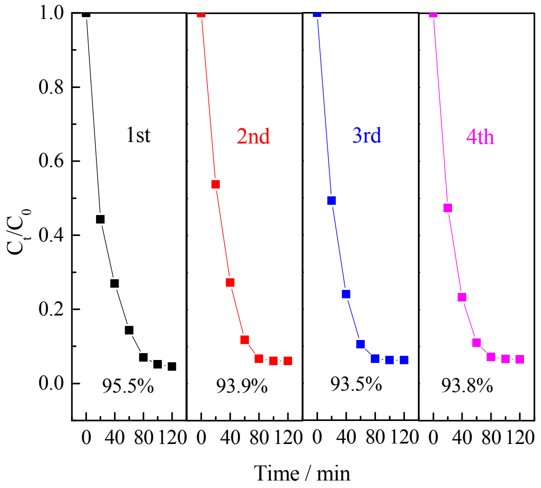

Figure S4. Recycle tests of Yb/TiO_2. The BHA concentration was 30 mg/L, and the catalyst dosage was 0.5 g/L.

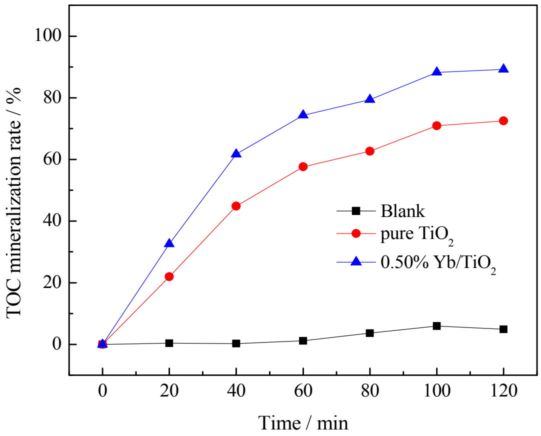

Figure S5. TOC (total organic carbon) efficiency by 0.50% Yb/TiO_2, pure TiO_2, and blank. The BHA concentration was 30 mg/L, and the catalyst dosage was 0.3 g/L.
